# Regenerative Endodontics in Teeth With Irreversible Pulpitis: A Scoping Review

**DOI:** 10.1111/iej.70179

**Published:** 2026-05-25

**Authors:** Mahmoud Torabinejad, Neda Najafimakhsoos, George Bogen

**Affiliations:** ^1^ Department of Endodontics School of Dentistry, Loma Linda University Loma Linda California USA; ^2^ Department of Biomedical and Neuromotor Sciences, Alma Mater Studiorum University of Bologna Bologna Emilia Romagna Italy; ^3^ Department of Endodontics School of Dentistry, University of Queensland Brisbane Queensland Australia

**Keywords:** immature teeth, irreversible pulpitis, mature teeth, mineral trioxide aggregate, pulp regeneration, pulp revitalisation, regenerative endodontics

## Abstract

**Introduction:**

Regenerative endodontic procedures (REPs) have been widely used to manage a range of pulpal pathologies, particularly in immature permanent teeth. The development of bioactive calcium silicate–based materials has led to a paradigm shift in vital pulp therapy (VPT) and REPs. Conventional clinical and radiographic criteria used to assess the healing ability of teeth diagnosed with irreversible pulpitis (IP) may be inadequate. Emerging evidence suggests that a proportion of pulps diagnosed as ‘irreversibly’ inflamed retain the capacity to recover normal function following VPT. The aim of this scoping review was to evaluate the current evidence supporting the possible use of REPs to preserve and regenerate vital pulp tissue in teeth diagnosed with IP.

**Methods:**

A comprehensive literature search was conducted using MEDLINE (via PubMed), Web of Science, the Cochrane Library, Google Scholar and Scopus to identify studies that met predefined inclusion and exclusion criteria. In addition, manual citation searching was undertaken to ensure comprehensive coverage of the relevant literature.

**Results:**

Four individual case reports describing moderately successful pulp regeneration using REPs in immature permanent teeth diagnosed with IP were identified. In addition, two case series and two case reports reported comparable outcomes following REPs in mature permanent teeth with the same pulpal diagnosis.

**Conclusions:**

Based on the results of this scoping review, the current clinical evidence supporting REPs for pulp regeneration in teeth diagnosed with IP is remarkably limited. However, biological plausibility is conceptually strong, and the potential focus of future investigations to achieve true pulp regeneration. Currently, VPT remains the preferable option for pulp preservation in teeth diagnosed with IP.

## 1 Introduction

Pulpitis represents an inflammatory response of the dental pulp to a variety of stimuli, including microbial invasion, chemical irritation, mechanical trauma and thermal injury (American Association of Endodontists 2020; Ricucci et al. 2014). Among these factors, dental caries, traumatic injuries and iatrogenic insults constitute the most significant aetiological challenges, owing to the extent and severity of damage inflicted on the dentine–pulp complex (Jiménez‐Martín et al. 2024; Xie et al. 2021). Clinically, pulpitis is classified as reversible or irreversible based on the presumed ability of the pulp to recover following elimination of the causative irritant (American Association of Endodontists 2020; Donnermeyer et al. 2023). In reversible pulpitis (RP), the pulp retains the capacity for repair and may return to a healthy state once the irritant is removed (Liu et al. 2025; Ricucci et al. 2014). In contrast, irreversible pulpitis (IP) is characterised by inflammatory changes that exceed the regenerative capacity of the pulp, ultimately progressing towards pulpal necrosis (Liu et al. 2025; Ricucci et al. 2014).

The clinical and radiographic diagnosis of IP is traditionally based on the nature, duration and intensity of symptoms (Baume 1970; Donnermeyer et al. 2023; Kumar et al. 2025). Diagnostic parameters commonly include responses to pulp sensibility testing, the presence and characteristics of pain (including spontaneity and persistence after stimulus removal), the time required to achieve haemostasis following caries excavation, and the radiographic depth of the carious lesion (Elmas et al. 2023; Zhu et al. 2013). Sensibility tests evaluate the functional integrity of sensory nerve fibres in response to thermal or electrical stimuli; however, they do not assess accurate pulp vitality, the degree of inflammation or the extent of microbial colonisation (Kahler et al. 2023; Lin et al. 2020). Importantly, a poor correlation exists between clinical symptoms, sensibility test responses and the actual severity of pulpal inflammation (Baume 1970; Dummer et al. 1980; Kahler et al. 2023; Lin et al. 2020). Although pain is frequently associated with pulpal infection and inflammation, it does not reliably reflect the underlying histopathological status of the pulp (Elmas et al. 2023; Kumar et al. 2025). Indeed, a previous study has demonstrated that approximately 40% of teeth presenting with pain harbour pulps that remain biologically salvageable (Elmas et al. 2023).

Similarly, the time required to achieve haemostasis after caries removal has been shown to be an unreliable indicator of pulpal status (Linsuwanont et al. 2017; Shah et al. 2025; Uesrichai et al. 2019). Achieving haemostasis within 10 min is an arbitrary number and does not definitively exclude the presence of infection (Alyahya and Qudeimat 2024; Duncan et al. 2019; Jassal et al. 2023; Lin et al. 2020; Uesrichai et al. 2019). Furthermore, neither the depth of the carious lesion nor the intensity or quality of pain reliably distinguishes RP from IP (Donnermeyer et al. 2023).

Histological assessment remains the most accurate method for determining the extent and severity of pulpal inflammation (Elmas et al. 2023; Kumar et al. 2025). Histologically, RP is characterised by a reduced number of fibroblasts, increased collagen fibre deposition and mild to moderate infiltration of lymphocytes and plasma cells, without disruption of the normal pulpal architecture (Ricucci et al. 2014). In contrast, IP is characterised by partial or complete necrosis of the coronal pulp, often accompanied by coagulative or liquefactive necrosis (Ricucci et al. 2014, 2019). These tissues typically exhibit dense infiltration of polymorphonuclear leukocytes, along with peripheral accumulation of chronic inflammatory cells, including lymphocytes, plasma cells and macrophages (Ricucci et al. 2014). A critical distinguishing feature between RP and IP is the presence of microbial colonisation, which is routinely observed in teeth with IP but is absent in healthy or reversibly inflamed pulps (Ricucci et al. 2014).

Histological studies have demonstrated that, in cariously exposed pulps, infection is often confined to the coronal pulp tissue (Kahler et al. 2023). Pulp tissue located only a few millimetres apical to the necrotic zone frequently appears healthy and free of inflammation or bacterial contamination (Lin et al. 2020; Taha et al. 2022). This cytological demarcation biologically distinguishes reversibly inflamed tissue from irreversibly inflamed coronal pulp (Ricucci et al. 2014, 2019). Consequently, removal of caries and amputation of the infected coronal pulp may establish a favourable biological environment that allows the remaining pulp tissue to recover and heal (Duncan et al. 2019; Jassal et al. 2023; Kahler et al. 2023; Lin et al. 2020). Notably, clinical diagnoses of RP do not correspond with histological findings in approximately 3.4% of cases, whereas discrepancies between clinical and histological diagnoses reach approximately 15.6% for IP (Ricucci et al. 2014). This suggests that a proportion of teeth clinically diagnosed with IP may, in fact, exhibit histological features consistent with RP (Ricucci et al. 2014).

Conventional root canal treatment (RCT) remains the standard of care for teeth diagnosed with IP (Ather et al. 2022; Shi et al. 2025). This approach involves complete removal of the pulp through mechanical instrumentation and chemical irrigation, followed by three‐dimensional obturation of the root canal system using gutta‐percha and endodontic sealers. Although RCT demonstrates high and predictable success rates (Doyle et al. 2006; Iqbal and Kim 2007; Ng et al. 2011; Torabinejad et al. 2007; Vahdati et al. 2019), preservation of vital pulp tissue warrants consideration in teeth presenting with clinical and radiographic signs suggestive of IP.

The introduction of mineral trioxide aggregate (MTA) as a pulp capping material resulted in a major advancement in the conservative management of IP in both immature and mature teeth (Pitt Ford et al. 1996). Vital pulp therapy (VPT), previously a traditional endodontic dogma in cases of symptomatic IP (Camp 2008), is now increasingly supported by clinical evidence when bioactive calcium silicate–based materials are employed (Taha et al. 2022). Historically, pulpotomy was seldom regarded as a definitive treatment for IP due to reliance on questionable diagnostic methods that assessed neural responsiveness as a surrogate for pulp vitality. However, advances in pulp biology, magnification technologies and the availability of biocompatible materials have caused a paradigm shift in VPT (Alyahya and Qudeimat 2024; Camp 2008; Duncan et al. 2019; Ricucci et al. 2019). Numerous studies have reported favourable clinical outcomes following caries removal and partial and complete pulpotomy in both immature and mature permanent teeth diagnosed with IP (Alyahya and Qudeimat 2024; Asgary et al. 2015; Elmas et al. 2023; Jiménez‐Martín et al. 2024; Sriudomdech et al. 2024; Taha et al. 2022; Taha and Abdelkhader 2018; Tzanetakis et al. 2023; Zhu et al. 2023), highlighting the remarkable regenerative capacity of inflamed pulp tissue (Alyahya and Qudeimat 2024; Louzada et al. 2025). VPT has become a relatively predictable, fundamentally simple, and cost‐effective option for pulp preservation in teeth diagnosed with IP.

The favourable outcomes reported following VPT are largely attributed to the excellent sealing ability and biocompatibility of contemporary calcium silicate–based materials and ability of dental pulp to generate mineralised repair tissues and heal (Parirokh et al. 2018). These bioactive cements promote the formation of thicker and more homogeneous mineralised tissue barrier. They induce less pulpal inflammation than traditional materials such as calcium hydroxide and allow dental pulp survival (Qudeimat et al. 2007; Shi et al. 2025). Importantly, after removal of infected coronal pulp tissue, so‐called ‘irreversibly inflamed’ pulp tissue can regain physiological equilibrium and functional capacity when adequately sealed with bioceramic materials (Louzada et al. 2025).

Experimental evidence further supports the repair and regenerative potential of residual pulp tissue. In an animal study, clot formation induced from a small volume of remaining, non‐inflamed apical pulp tissue created a biological environment conducive to complete regeneration following deep pulpotomy using a modified regenerative endodontic technique (Torabinejad et al. 2018). This animal study reinforces the biological rationale for preserving vital tissue when possible. Given that conversion of a clinical diagnosis of IP to a biologically reversible state appears possible following pulpotomy with bioceramic materials, it may be hypothesised that pulp tissue regeneration could serve as a possible treatment option following pulpotomy in teeth diagnosed with IP under proper conditions. A potential advantage of this approach is preserving remaining vital pulp and its use as a resource for regeneration of a greater volume of pulp tissue, or even major portions of the entire pulp.

Regenerative endodontic procedures (REPs) apply the principles of tissue engineering to pulp therapy and encompass a variety treatment protocols. These can include pulp revitalisation, pulp revasculatization, and pulp regeneration (PR) (Galler 2016; Nunes et al. 2026). The American Association of Endodontists defines regenerative endodontics as biologically based procedures designed to regenerate the dentine–pulp complex (American Association of Endodontists 2021). When regeneration is achieved through induction of bleeding and endogenous cell recruitment from periradicular tissues, the process is not revascularisation or PR as previously proposed (Banchs and Trope 2004), but instead considered ‘revitalization’ (Galler 2016). This approach has been typically applied in immature teeth, where stem cells from the apical papilla (SCAP) and periapical tissues play a central role in tissue regeneration (Ahmed et al. 2021; Duncan et al. 2018; Lovelace et al. 2011). In mature teeth, revitalisation procedures are more challenging due to the absence of SCAP and the presence of a narrower apical foramen, which may limit cellular ingress and vascularisation (Duncan et al. 2018; Huang et al. 2008; Lovelace et al. 2011).

Two principal biological strategies have been proposed to facilitate pulp tissue regeneration within the root canal system: cell‐based transplantation and cell homing. Cell‐based approaches involve the delivery of exogenous stem cells into the canal space, whereas cell homing strategies rely on recruitment of endogenous stem cells using appropriate scaffolds and signalling molecules (Ahmed et al. 2021; Shi et al. 2025).

A substantial body of evidence supports the use of REPs in immature permanent teeth with necrotic pulps, demonstrating favourable clinical and radiographic outcomes, including resolution of symptoms, continued root development and apical closure (Banchs and Trope 2004; Iwaya et al. 2001; Jeeruphan et al. 2012; Lenzi et al. 2022; Nagy et al. 2014; Silujjai and Linsuwanont 2017; Theekakul et al. 2024; Torabinejad and Turman 2011; Wigler et al. 2013; Zhu et al. 2013). REPs have also been successfully applied in mature teeth for the management of traumatic injuries and root resorption (Kaval et al. 2018; Lu and Kahler 2022; Saoud et al. 2016; Yoshpe et al. 2020).

However, the application of REPs for the treatment of residual, non‐infected inflamed pulp tissue in teeth diagnosed with IP has not been systematically evaluated as an ancillary or alternative to partial or complete pulpotomy. Given the emerging biological evidence supporting pulp healing and its regeneration potential following removal of infected tissues, this represents an important knowledge gap. Accordingly, a scoping review was selected as the most appropriate methodological approach to map the existing evidence for the use of REPs in teeth with immature and mature apices diagnosed with IP. This approach allows inclusion of diverse study designs with heterogeneous protocols, facilitates identification of knowledge gaps, and enables synthesis of preliminary findings to guide future research and clinical investigations. Therefore, the aim of this scoping review was to evaluate the current evidence for the possible application of REPs in teeth diagnosed with IP.

## 2 Methods

This scoping review was conducted in accordance with the Preferred Reporting Items for Systematic Reviews and Meta‐Analyses extension for Scoping Reviews (PRISMA‐ScR) guidelines (Tricco et al. 2018). The review protocol was developed following consultation with an experienced health sciences librarian and through iterative discussion among the review team to refine the research question, eligibility criteria and search strategy. This study has been registered in the Open Science Framework. The registration record is available at DOI: https://doi.org/10.17605/OSF.IO/R6JBS.

### 2.1 Search Strategy

A comprehensive electronic literature search was performed to identify relevant studies published up to October 2025. The following databases were searched: MEDLINE (via PubMed), Web of Science, the Cochrane Library, Google Scholar and Scopus. Owing to the limited capacity of Google Scholar for structured systematic searches, only the first 100 results for each search query were screened to ensure feasibility while maintaining relevance. In addition, a manual search of reference lists from relevant articles and reviews was undertaken to identify additional eligible studies not captured through electronic database searching.

The search strategy combined free‐text keywords with Medical Subject Headings (MeSH), where applicable. The following terms and their various combinations were used:

- Vital pulp therapy
- Regenerative endodontics
- Pulp revascularisation
- Dental pulp regeneration
- Reversible pulpitis
- Irreversible pulpitis
- Immature and mature teeth
- Open and closed apices
- Necrotic pulp
- Periapical lesions
- Endodontic treatment using stem cells
- Tissue engineering in pulp regeneration

**FIGURE 1:**
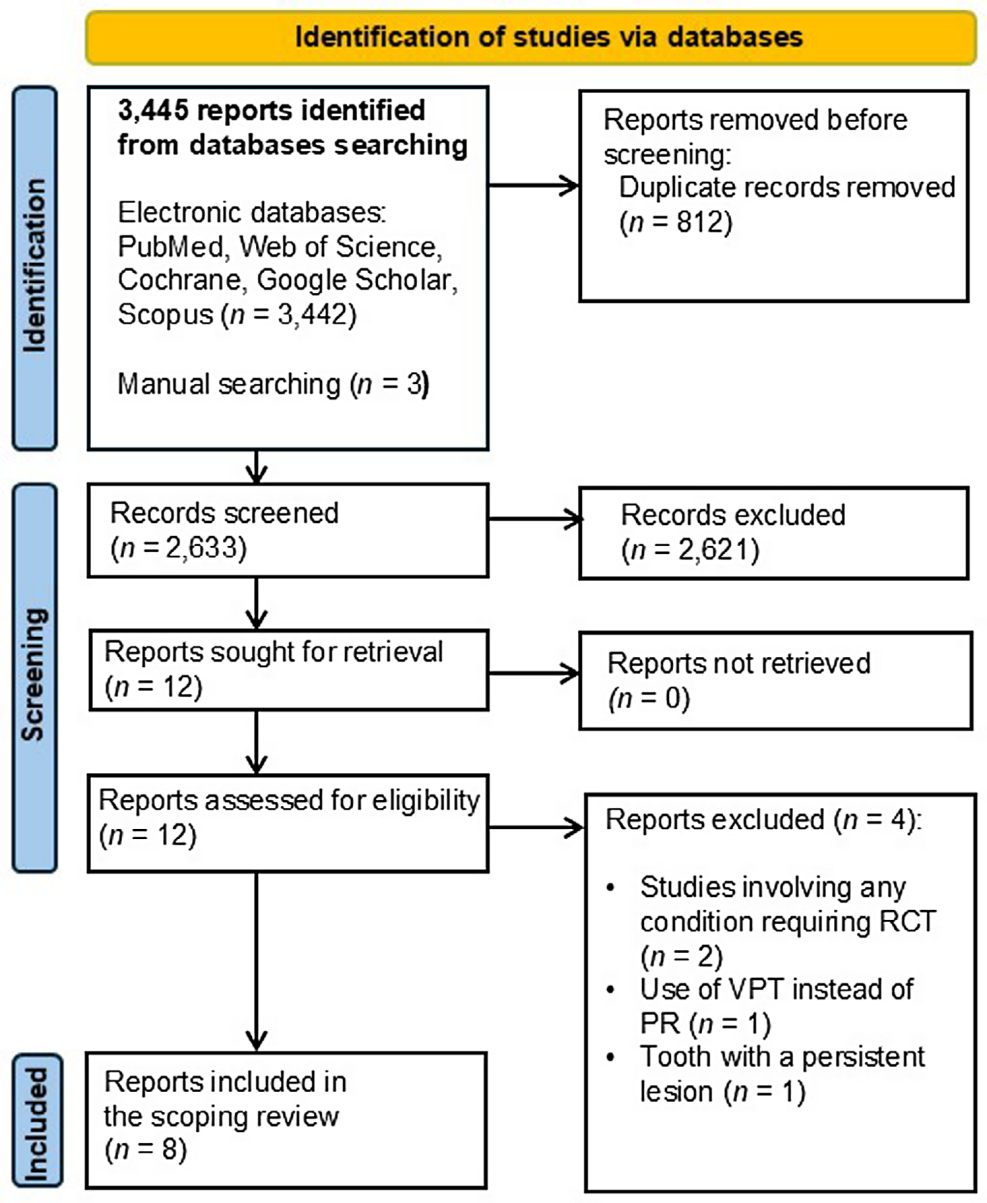
Flow diagram illustrating study identification, screening and inclusion based on predefined eligibility criteria.

The titles and abstracts of all retrieved records were independently screened by two reviewers to assess eligibility. Full‐text articles were subsequently evaluated for inclusion based on predefined criteria. The study selection process is illustrated in Figure 1.

### 2.2 Eligibility Criteria

#### 2.2.1 Inclusion Criteria

Studies published in English that evaluated pulp regenerative procedures in immature or mature permanent teeth diagnosed with irreversible pulpitis were included. Eligible study designs comprised case reports, case series and pilot clinical studies. To maximise capture of relevant evidence, all studies meeting the inclusion criteria were considered regardless of publication year, up to October 2025.

#### 2.2.2 Exclusion Criteria

Animal studies, in vitro investigations, studies involving primary teeth and studies in which pulp necrosis was the primary diagnosis were excluded. Grey literature, including theses, conference abstracts and unpublished studies, were also excluded.

### 2.3 Study Selection and Data Charting

Two reviewers independently screened titles, abstracts and full texts for eligibility. Data charting was conducted independently using a predefined data extraction framework. Any disagreements regarding study selection or extracted data were resolved through discussion between the two reviewers, and when necessary, by consultation with a third reviewer. No attempts were made to contact study authors for additional information, as most relevant data required for this review was available within the published reports.

### 2.4 Data Synthesis

Charted data were organised into two tables: one summarising reports involving immature permanent teeth and the other summarising reports involving mature permanent teeth. Key characteristics and outcomes of each included report were also synthesised narratively. Owing to substantial heterogeneity in study design, outcome measures and the limited number of available reports, quantitative synthesis and meta‐analysis were not feasible.

## 3 Results

### 3.1 Study Selection

The electronic database search identified a total of 3442 records, with a further three reports identified through manual reference searching, resulting in 3445 records overall. After removal of 812 duplicate records, 2633 titles and abstracts were screened for relevance. Of these, 2621 records were excluded as they did not meet the inclusion criteria. Twelve full‐text articles were assessed for eligibility. Following full‐text review, four reports were excluded for the predefined reasons (Feitosa et al. 2021; Jafari et al. 2025; Kim et al. 2025; Ramezani et al. 2019) (Table 1). Consequently, eight reports were included in the final qualitative synthesis (Das et al. 2023; Lu and Kahler 2022; Meza et al. 2019; Nakashima et al. 2017, 2022; Peng et al. 2017; Sabeti et al. 2021; Shimizu et al. 2012) (Tables 2 and 3). The study selection process is illustrated in Figure 1.

**TABLE 1:** Studies excluded after full‐text assessment and reasons for exclusion.

Study	Year	Reason for exclusion
Feitosa et al.	2021	Included teeth had periapical lesions and were not diagnosed with irreversible pulpitis.
Jafari et al.	2025	Treated teeth presented with persistent periapical radiolucency and widening of the periodontal ligament space on radiographic examination.
Kim et al.	2025	Included any tooth condition requiring RCT.
Ramezani et al.	2019	Revascularization is used in teeth with necrotic pulps while vital pulp therapy was used for teeth with IP.

**TABLE 2:** Characteristics of studies reporting regenerative endodontic procedures in immature permanent teeth diagnosed with irreversible pulpitis.

Study	Journal	Study type	No. of teeth	Type of tooth	Age	M/F	RPT	Bioceramic cement	Methods of evaluation				Clinical test		Follow‐up (months)	Country
									Histology	Immuno	RX	Laser	Cold	EPT		
Das et al. 2023	Cureus	Case Report	1	Mandibular First Molar	7	1 M	CH	White MTA	No	No	Periapical, OPG	No	−	−	3–6	India
Peng et al. 2017	JOE	Case Report	1	Mandibular Second Premolar	11	1F	CH	No, Fuji IX Glass Ionomer Cement	Yes	No	Periapical	No	−	−	3–12–24	China
Sabeti et al. (2021)	JOE	Case Report	1	Maxillary Central Incisor	9	1F	CH	ProRoot White MTA	No	No	CBCT Periapical	No	+	+	6–12–18	USA
Shimizu et al. (2012)	JOE	Case Report	1	Maxillary Central Incisor	10	1 M	CH	ProRoot Mineral trioxide aggregate (MTA)	Yes	Yes	No	No	NR	NR	Less than 1 month	USA

Abbreviations: CBCT, cone beam computed tomography; CH, cell homing; CT, cell transplantation; EPT, electric pulp test; F, female; Immuno., immunohistochemical; M, male; M/F, male/female; MTA, mineral trioxide aggregate; N, number; NR, not reported; OPG, orthopantomogram; RPT, regenerative protocol type, RX, radiography.

**TABLE 3:** Characteristics of studies reporting regenerative endodontic procedures in mature permanent teeth diagnosed with irreversible pulpitis.

Study	Journal	Study type	No. of teeth	Type of tooth	Age	M/F	RPT	Stem cell	Scaffold	Growth factor	Bioceramic cement	Methods of evaluations				Clinical test		Follow‐up (months)	County
												Histology	Immuno	RX	Laser	Cold	EPT		
Lu and Kahler (2022)	BMC oral health	Case report	2	Maxillary central incisors	17	0 M/1F	CH	Endogenous	Blood clot	Endogenous	iRoot BP	No	No	CBCT Periapical	No	+	+	1–3–6–12–14–24–25–36–48	China Australia
Meza et al. (2019)	JOE	Case report	1	Mandibular first premolar	50	1 M	CT	DPSCs	L‐PRF	L‐PRF	Biodentine (Septodont)	No	No	CBCT; Periapical	Yes	+	+	6–36	Chile
Nakashima et al. (2017)	Stem Cell Res Ther	Case series	5	Premolars; incisors	20–44	3 M/2F	CT	MDPSCs	Atelo collagen	G‐CSF	No, GC Fuji IX Extra Glass Ionomer	No	No	Periapical; MRI; CBCT	No	NR	+	1–3–6–7–8–9	Japan
Nakashima et al. (2022)	JOE	Case series	2	Maxillary molars	26–29	2 M/0F	CT	Autologous hDPSCs from extracted third molar	Atelo collagen	G‐CSF	Biodentine (Septodont)	No	No	Periapical; MRI; CBCT	No	Init.−; NR	+	1–3–6–12	Japan

Abbreviations: CBCT, cone beam computed tomography; CH, cell homing; CT, cell transplantation; DPSCs, dental pulp stem cells; EPT, electric pulp test; F, female; G‐CSF, granulocyte colony stimulating factor; hpDPSCs, hypoxic DPSCs; Immuno., immunohistochemical; Init., initially; L‐PRF, leukocyte platelet‐rich fibrin; M, male; MDPSCs, mobilised dental pulp stem cells; M/F, male/female; MRI, magnetic resonance imaging; MTA, mineral trioxide aggregate; N, number; NR, not reported; RPT, regenerative protocol type; RX, radiography.

### 3.2 Regenerative Endodontic Procedures in Immature Permanent Teeth

Four case reports described the use of REPs in immature permanent teeth diagnosed with IP (Table 2). In the first report, REPs were performed in a permanent tooth with an open apex, followed by histological and immunohistochemical evaluation after 3.5 weeks (Shimizu et al. 2012). The authors reported the presence of immature pulp‐like connective tissue containing mesenchymal cells, abundant blood vessels in the apical region and flattened odontoblast‐like cells lining the predentine.

In the second case report, outcomes were assessed at 3, 12 and 24 months following REPs (Peng et al. 2017). The tooth remained asymptomatic and demonstrated radiographic evidence of continued root maturation, although it did not respond to pulp sensibility testing. Histological findings in this case were comparable to those reported by Shimizu et al. (2012).

The third case report involved two symptomatic traumatised maxillary central incisors with open apices both treated with REPs (Sabeti et al. 2021). The right central incisor was diagnosed with a necrotic pulp and the left central incisor exhibited signs and symptoms consistent with IP. The treated left central incisor diagnosed with IP displayed positive responses to cold and electric pulp testing, absence of clinical symptoms and radiographic evidence of continued root development at 6‐, 12‐ and 18‐month follow‐up periods.

In the fourth case report, REPs were performed using platelet‐rich fibrin as a scaffold in an immature permanent tooth (Das et al. 2023). Continued root development and absence of clinical symptoms were observed at 3 and 6 months post‐treatment; however, the tooth did not respond to pulp sensibility tests.

### 3.3 Regenerative Endodontic Procedures in Mature Permanent Teeth

Two case series and two case reports described REPs performed in mature permanent teeth diagnosed with IP (Table 3). In the first case series, regenerative procedures were performed in five mature permanent teeth using autologous mobilised dental pulp stem cells (MDPSCs) combined with granulocyte colony‐stimulating factor (G‐CSF) (Nakashima et al. 2017). Outcomes were assessed using periapical radiography, magnetic resonance imaging (MRI), cone beam computed tomography (CBCT) and electric pulp testing at follow‐up intervals ranging from 1 to 9 months. Four teeth demonstrated positive responses to electric pulp testing within 1 month, while one tooth responded positively at 9 months. CBCT imaging revealed dentine‐like tissue thickening of the lateral canal walls in three cases, and MRI demonstrated high signal intensity in the apical and coronal regions of four teeth, suggestive of viable regenerated pulp tissue.

In a subsequent case series, hypoxic dental pulp stem cells combined with G‐CSF were used in two mature permanent teeth, with follow‐up extending to 48 weeks (Nakashima et al. 2022). Both teeth remained asymptomatic and responded positively to electric pulp testing. CBCT imaging demonstrated mineralised tissue formation in the apical third of the canal and dentine‐like tissue deposition along the lateral canal walls. Another case report described the use of dental pulp stem cells combined with leukocyte platelet‐rich fibrin in a mature permanent tooth (Meza et al. 2019). Clinical and radiographic outcomes were assessed using periapical radiographs, CBCT, electric pulp testing, cold testing and laser Doppler flowmetry at 6 and 36 months. The tooth remained asymptomatic, exhibited normal mobility and responded positively to sensibility testing. CBCT imaging revealed intact periapical bone, a mineralised dentine‐like bridge in the middle third of the canal and calcification in the apical third. Laser Doppler flowmetry confirmed pulpal blood flow without adverse findings.

In the final case report, REPs were performed in two mature permanent teeth with transverse root fractures (Lu and Kahler 2022). Clinical and radiographic follow‐up over 48 months demonstrated absence of symptoms, delayed positive responses to cold and electric pulp testing and calcified tissue formation along the fracture lines. CBCT imaging confirmed healing through fibrous connective tissue formation and realignment of the fractured segments.

## 4 Discussion

The principal objectives of ‘pulp regeneration’ include resolution of clinical signs and symptoms, radiographic healing of apical periodontitis when present, and recovery of pulpal sensibility and physiological function (Ahmed et al. 2021; Shi et al. 2025). In immature permanent teeth, additional therapeutic goals include continued root development and apical closure (Murray 2023; Sabeti et al. 2021; Shi et al. 2025). From a histological perspective, successful PR is characterised by the presence of connective tissue resembling native pulp, neovascularisation, reinnervation and deposition of tubular dentine‐like tissue along the canal walls, with an organised layer of odontoblast‐like cells lining the newly formed mineralised matrix (Ahmed et al. 2021; Shi et al. 2025). However, the tissues formed within the root canal space following conventional REPs do not consistently represent authentic regeneration of the dentine–pulp complex. Animal studies (Altaii et al. 2017; Dos Reis‐Prado et al. 2023; Palma et al. 2017; Torabinejad et al. 2014, 2018) and several human histological investigations (Lui et al. 2020; Martin et al. 2013; Nosrat et al. 2015; Shimizu et al. 2013) have demonstrated that the canal space is frequently occupied by connective tissue, cementum or bone rather than pulp tissue. This phenomenon is largely attributed to blood clot scaffolds induced by bleeding from periradicular tissues, which facilitates homing of periodontal ligament fibroblasts, cementoblasts and osteogenic cells into the disinfected canal space (Torabinejad et al. 2018). Consequently, such outcomes should be accurately described as functional repair rather than true regeneration.

The term revitalisation more accurately describes this biologically based approach aimed at reestablishing vital tissue within the root canal system following disinfection with induction of bleeding and blood clot formation (Galler 2016). An animal study using immature dog teeth was conducted in which apical periodontitis was experimentally induced by placing supragingival plaque in the access cavities (Palma et al. 2017). After 3 weeks, radiographic confirmation of apical lesions was obtained and three protocols were evaluated using REPs; blood clot only, sodium hyaluronate–chitosan, and pectin‐chitosan scaffolds. After 13 weeks, histology showed all groups formed mainly vascularized connective tissue with ectopic calcifications and cementum‐like deposits, not innate pulp. Only the blood clot group exhibited dentine formation with odontoblast‐like cells, indicating limited pulp–dentine complex regeneration in 21% of specimens. The origin of the odontoblast‐like cells was unclear, as original odontoblasts may have survived and contributed to continued tubular dentine formation rather than newly differentiated dental pulp stem cells. Observations from this study indicated blood clot scaffolds were superior to artificial scaffolds in reforming lost pulp tissues. Additionally, the mechanical instrumentation that induces haemorrhage from periapical tissues may also compromise SCAP function and root development. Furthermore, histological evidence also confirmed previous findings that newly formed mineralised hard tissues are separate from the original immature root dentine and not integrated, questioning claims of actual wall thickening and increased root strength (Lui et al. 2020).

REPs have become widely accepted as a biologically based alternative to traditional apexification techniques using calcium hydroxide or MTA apical barriers in immature teeth with necrotic pulps (Lenzi et al. 2022; Murray 2023; Nagy et al. 2014; Torabinejad and Turman 2011; Zhu et al. 2013). Several studies have reported significantly greater increases in root length and thickness following REPs compared with apexification procedures (Caleza‐Jiménez et al. 2022; Cheng et al. 2025; Theekakul et al. 2024). Beyond necrotic immature teeth, this procedure also has demonstrated favourable outcomes in the management of traumatic injuries, root perforations, avulsion injuries and both internal and external root resorption in mature and immature teeth (Cheng et al. 2025; Kaval et al. 2018; Loroño et al. 2022; Lu and Kahler 2022; Saoud et al. 2016; Yoshpe et al. 2020). A recent systematic review further reported successful arrests of root resorption and resolution of clinical symptoms following REPs, with a concomitant reduction in periapical pathology (Dadpe et al. 2023).

Only two human case reports have provided histological evidence of pulp‐like tissue regeneration following REPs (Shimizu et al. 2012; Torabinejad and Faras 2012). These results might have been achieved due to the survival of the original odontoblasts rather than newly differentiated dental pulp stem cells. However, the promising results in mature teeth using cell transplantation have not yet been reported histologically (Lu and Kahler 2022; Meza et al. 2019; Nakashima et al. 2017, 2022). In contrast, genuine PR has been demonstrated histologically in an animal model using immature ferret canines. After access, pulp tissue was removed in which a small volume (approximately 1–4 mm) of viable, non‐inflamed apical pulp tissue was intentionally preserved without sodium hypochlorite or antibiotic paste sterilisation of the canal walls. In this model, PR was induced by blood clot scaffolds from the residual intracanal pulp tissue rather than elements from periapical tissues by mechanical instrumentation beyond the apex (Torabinejad et al. 2018). The resulting blood clot appeared to act as a biological scaffold while the preserved apical pulp served as a barrier preventing ingress of periradicular cells and tissues. This approach resulted in the formation of pulp‐like connective tissue with odontoblast‐like cells lining the dentinal walls, whereas controls with complete pulp removal led to ectopic bone formation within the canal space (Torabinejad et al. 2018).

These findings highlight the critical importance of preserving viable, uninfected pulp tissue as the source of haemorrhage and clot formation to facilitate the potential for authentic regeneration of the dentine–pulp complex. Preservation of residual pulp tissue not only supports formation of pulp‐like connective tissue but also promotes differentiation of odontoblast‐like cells along the canal periphery and deposition of dentine‐like mineralised tissue beneath the bioceramic interface (Torabinejad et al. 2018). This is true PR that refers to the restoration of the dental pulp to its original condition, architecture and function. This includes odontoblast and pulp‐like tissue formation within the root canal, where differentiation of endogenous cells into odontoblasts capable of secreting tubular dentine occurs (Galler 2016; Galler et al. 2016). This novel antithetical approach to produce blood clot scaffolds from remaining vital pulp tissues can change accepted REP paradigms and redirect research to achieve the ultimate threshold of genuine pulp tissue generation.

The four case reports involving immature teeth included in this scoping review provide limited preliminary evidence for preserving the apical portion of the pulp and performing REPs in teeth diagnosed with IP (Das et al. 2023; Peng et al. 2017; Sabeti et al. 2021; Shimizu et al. 2012). Only two out of four of these reports presented histological findings (Peng et al. 2017; Shimizu et al. 2012). In these cases, the fundamental components of tissue engineering, namely a cell source, scaffold and signalling molecules—appeared to be inherently present within the preserved pulp tissue (Hargreaves et al. 2013). Although pulp tissue in teeth diagnosed with IP is typically inflamed, putative stem cell populations with proliferative and differentiation capacity have been identified within such tissues (Wang et al. 2010). Moreover, the stromal cell‐derived factor‐1 (SDF‐1)/CXC chemokine receptor 4 (CXCR4) axis, which plays a key role in stem cell recruitment following tissue injury, is upregulated in inflamed dental pulp, suggesting that inflammation itself may facilitate endogenous stem cell homing (Abbott et al. 2004; Jiang, Ling, and Gong 2008; Jiang, Zhu, et al. 2008; Wang et al. 2010). Collectively, these observations suggest that removal of infected coronal pulp tissue may permit the remaining pulp to recover and regenerate when protected from external microbial irritation and sealed with bioceramic materials (American Association of Endodontists 2020; Shi et al. 2025).

The application of REPs in mature permanent teeth, traditionally considered less favourable candidates for ‘regenerative therapy’, has also been explored with encouraging preliminary outcomes. Case reports, systematic reviews and early clinical trials have reported reduced postoperative symptoms and favourable periapical healing following regenerative procedures in mature teeth with necrotic pulps (Arslan et al. 2019; Glynis et al. 2021; Paryani and Kim 2013; Scelza et al. 2021; Youssef et al. 2022). A randomised clinical trial reported fewer postoperative symptoms during the first 12 h following regenerative treatment compared with conventional root canal treatment, with comparable reductions in periapical lesion size (Ahmed et al. 2023). Additional studies have reported favourable healing outcomes using platelet‐rich fibrin and concentrated growth factors in mature necrotic teeth (Salah et al. 2025).

Within the present scoping review, two case series and two case reports described ‘pulp generation’ in mature permanent teeth diagnosed with IP (Lu and Kahler 2022; Meza et al. 2019; Nakashima et al. 2017, 2022). Three of these reports employed cell‐based transplantation strategies, incorporating autologous dental pulp stem cells, growth factors and bioactive scaffolds (Meza et al. 2019; Nakashima et al. 2017, 2022). Resolution of symptoms and recovery of pulp sensibility were observed in most cases; however, histological confirmation of regenerated pulp tissue was not available. Moreover, follow‐up periods varied considerably, with only two studies reporting outcomes beyond three years (Lu and Kahler 2022; Meza et al. 2019). Unlike REPs in immature teeth, the essential components required for tissue engineering in mature teeth were largely introduced exogenously rather than relying on endogenous elements needed for pulp tissue regeneration. The promising results in mature teeth using cell‐transplantation suffer from protocol heterogeneities and have not yet provided similar histological confirmation observed in immature teeth (Peng et al. 2017; Shimizu et al. 2012).

The findings of the present scoping review highlight the insufficient volume of evidence regarding pulp tissue regeneration in teeth diagnosed with IP. The included studies were primarily case reports and small case series, which inherently provide a low level of clinical evidence and limit the ability to draw definitive conclusions regarding the effectiveness of this approach. Moreover, the diverse study designs, differences in biological approaches and varying assortment of biomaterials used in these case reports underscore the experimental nature of REPs in treating inflamed pulps (Palma and Nascimento 2026). In contrast, the evidence supporting VPT as a viable treatment option for pulp preservation is substantial as clinical acceptance increases and treatment complexity is reduced when compared to REPs (Hatipoğlu et al. 2023). Although more established areas of regenerative endodontics, such as revitalization procedures in necrotic teeth, are supported by an exceptionally stronger evidence base, clinical application of REPs are still primarily performed by endodontic specialists (Pertek Hatipoğlu et al. 2025). A current systematic review and meta‐analysis synthesised data from multiple clinical studies provided quantitative evidence revealing that the effectiveness of current regenerative protocols in teeth with apical periodontitis is not supported when compared to fully pulpectomised teeth (Meschi et al. 2023). Similarly, comprehensive bibliometric analysis has demonstrated that clinical recommendations in regenerative endodontics are typically supported by higher levels of evidence. However, the absence of reliable standardised protocols weakens the acceptance of REPs due to the heterogenicity of available evidence‐based research (Nunes et al. 2026). Compared with these more robust bodies of evidence, the current literature addressing REPs in teeth with IP remains sparse and predominantly exploratory.

## 5 Conclusion

While current reports suggest histological feasibility of pulp tissue regeneration in teeth diagnosed with IP, evidence remains insufficient for definitive clinical recommendations. However, the biological plausibility is strong and does support further research utilising blood clot scaffolds of pulp tissue origin shifting REP paradigms. Well‐designed prospective and randomised studies with larger samples, standardised protocols, and long‐term follow‐up are needed to assess the potential for true PR and its predictability. Currently, VPT remains the preferable option for pulp preservation in teeth with IP.

## Author Contributions

**Mahmoud Torabinejad:** conceptualization; data extraction; methodology; validation; writing – review and editing. **Neda Najafimakhsoos:** conceptualization; data extraction; data visualisation; formal analysis; validation; writing – original draft; and Writing – review. **George Bogen:** data extraction; validation; writing – review and editing.

## Funding

The authors have nothing to report.

## Conflicts of Interest

Mahmoud Torabinejad currently has no conflicts of interest with the subject matter or materials discussed in this manuscript. However, he has previously received royalties as an inventor of several products through Loma Linda University. The remaining authors declare no conflicts of interest.

## Data Availability

The data that supports the findings of this review are available upon reasonable request from the corresponding author.
